# Cardiovascular events among men with prostate cancer treated with androgen receptor signaling inhibitors: a systematic review, meta-analysis, and network meta-analysis

**DOI:** 10.1038/s41391-024-00886-0

**Published:** 2024-09-05

**Authors:** Akihiro Matsukawa, Takafumi Yanagisawa, Mehdi Kardoust Parizi, Ekaterina Laukhtina, Jakob Klemm, Tamás Fazekas, Keiichiro Mori, Shoji Kimura, Alberto Briganti, Guillaume Ploussard, Pierre I. Karakiewicz, Jun Miki, Takahiro Kimura, Pawel Rajwa, Shahrokh F. Shariat

**Affiliations:** 1 Department of Urology, Comprehensive Cancer Center, Medical University of Vienna, Vienna, Austria; 2 Department of Urology, The Jikei University School of Medicine, Tokyo, Japan; 3 Department of Urology, Shariati Hospital, Tehran University of Medical Science, Tehran, Iran; 4 Department of Urology, University Medical Center Hamburg-Eppendorf, Hamburg, Germany; 5 Department of Urology, Semmelweis University, Budapest, Hungary; 6 Unit of Urology, Urological Research Institute, IRCCS San Raffaele Scientific Institute, Milan, Italy; 7 Vita-Salute San Raffaele University, Milan, Italy; 8 Department of Urology, La Croix du Sud Hospital, Quint Fonsegrives, France; 9 Cancer Prognostics and Health Outcomes Unit, Division of Urology, University of Montréal Health Center, Montréal, QC Canada; 10 Department of Urology, Medical University of Silesia, Zabrze, Poland; 11 Department of Urology, University of Texas Southwestern Medical Center, Dallas, TX USA; 12 Department of Urology, Weill Cornell Medical College, New York, NY USA; 13 Department of Urology, Second Faculty of Medicine, Charles University, Prague, Czechia; 14 Division of Urology, Department of Special Surgery, The University of Jordan, Amman, Jordan; 15 Karl Landsteiner Institute of Urology and Andrology, Vienna, Austria; 16 Research Center for Evidence Medicine, Urology Department Tabriz University of Medical Sciences, Tabriz, Iran

**Keywords:** Cancer therapy, Outcomes research

## Abstract

**Background:**

Androgen-receptor pathway inhibitors (ARPIs) have dramatically changed the management of advanced/metastatic prostate cancer (PCa). However, their cardiovascular toxicity remains to be clarified.

**Objective:**

To analyze and compare the risks of cardiovascular events secondary to treatment of PCa patients with different ARPIs.

**Methods:**

In August 2023, we queried PubMed, Scopus, and Web of Science databases to identify randomized controlled studies (RCTs) that analyze PCa patients treated with abiraterone, apalutamide, darolutamide, and enzalutamide. The primary outcomes of interest were the incidence of cardiac disorder, heart failure, ischemic heart disease (IHD), atrial fibrillation (AF), and hypertension. Network meta-analyses (NMAs) were conducted to compare the differential outcomes of each ARPI plus androgen deprivation therapy (ADT) compared to standard of care (SOC).

**Results:**

Overall, 26 RCTs were included. ARPIs were associated with an increased risk of cardiac disorders (RR: 1.74, 95% CI: 1.13–2.68, *p* = 0.01), heart failure (RR: 2.49, 95% CI: 1.05–5.91, *p* = 0.04), AF (RR: 2.15, 95% CI: 1.14–4.07, *p* = 0.02), and hypertension (RR: 2.06, 95% CI: 1.67–2.54, *p* < 0.01) at grade ≥3. Based on NMAs, abiraterone increased the risk of grade ≥3 cardiac disorder (RR:2.40, 95% CI: 1.42–4.06) and hypertension (RR:2.19, 95% CI: 1.77–2.70). Enzalutamide was associated with the increase of grade ≥3 AF(RR: 3.17, 95% CI: 1.05–9.58) and hypertension (RR:2.30, 95% CI: 1.82–2.92).

**Conclusions:**

The addition of ARPIs to ADT increases the risk of cardiac disorders, including IHD and AF, as well as hypertension. Each ARPI exhibits a distinct cardiovascular event profile. Selecting patients carefully and vigilant monitoring for cardiovascular issues is imperative for those undergoing ARPI + ADT treatment.

## Introduction

The introduction of androgen-receptor pathway inhibitors (ARPIs), including abiraterone acetate, apalutamide, darolutamide, and enzalutamide has significantly transformed the treatment landscape for advanced/metastatic prostate cancer (PCa) based on definitive survival benefits when added to androgen deprivation therapy (ADT) [1, 2]. While these agents prolong overall survival (OS) [3–15], the increased duration of treatment necessitates careful selection of an appropriate ARPI, taking into account its safety profile to decrease potential adverse events (AEs). Furthermore, the type, rate, and severity of AEs are affected by the patient’s general health state and disease state (i.e., non-metastatic castration-resistant prostate cancer [nmCRPC], metastatic castration-resistant prostate cancer [mCRPC], metastatic/advanced hormone-sensitive prostate cancer [HSPC], biochemical recurrence [BCR]), requiring a detailed assessment of each patient prior to the selection of the optimal ARPI.

In general, all ARPIs exhibit acceptable tolerability and safety profiles in randomized controlled trials (RCTs) with an acceptable rate of treatment discontinuation. A meta-analysis showed that the incidence of AEs in mHSPC patients treated with ARPI and ADT is not significantly different from those caused by standard of care (SOC) [1]. However, data comparing system-specific types of AEs of ARPIs and AE incidence across different PCa states are limited. Cardiac disorders, which are the AEs with the highest potential for lethality, have been reported in approximately 6–23% [4, 5, 9, 11, 12] of patients treated with ARPI plus ADT across RCTs. The importance of managing non-negligible cardiovascular events during ADT has been a subject of treatment selection [16]. However, there is no comprehensive data synthesizing the impact of ARPIs plus ADT and specific types of ARPIs on the risk of subsequent cardiovascular disease in PCa patients treated with ARPIs. This would have a substantial impact on shared decision-making, especially for patients with a long survival probability due to the cumulative risk (i.e., mHSPC patients with low-volume disease, slow progression nmCRPC patients, or BCR patients). Therefore, we conducted this meta-analysis to comprehensively assess the impact of ARPIs on the risk of cardiovascular events and compare the differential outcomes based on different ARPIs across all PCa states (i.e., nmCRPC, mCRPC, metastatic/advanced HSPC, and BCR).

## Methods

Our study protocol is registered with the International Prospective Register of Systemic Reviews database (**PROSPERO: CRD42023452885**). This meta-analysis adheres to the guidelines of the Preferred Reporting Items for Systematic Reviews and Meta-Analyses (PRISMA) statement and AMSTAR2 checklist [17, 18].

### Study selection and characteristics

A literature search was conducted in August 2023 using the PubMed, Scopus, and Web of Science databases to identify studies that investigated the incidence of cardiovascular events associated with ARPI for advanced PCa. The comprehensive search strategy is detailed in Supplementary Appendix 1. The primary outcome of interest was the incidence of cardiovascular events. Initial screening based on the titles and abstracts was performed by two investigators to identify eligible studies. Studies deemed potentially relevant underwent a full-text review. Disagreements were settled by consensus with co-authors.

### Inclusion and exclusion criteria

All RCTs assessing the AEs of ARPI, such as abiraterone, enzalutamide, apalutamide, and darolutamide are included. We utilized the PICO framework [19]. Included studies must have evaluated patients with metastatic/advanced HSPC, nmCRPC, mCRPC, or BCR (Population), treated with ARPI plus ADT (Intervention), and compared to those treated with SOC (Comparison) to assess the risk of cardiovascular events (Outcome). The primary outcome of interest was the overall proportion of cardiac disorders, which are defined according to the National Cancer Institute’s Common Terminology Criteria for Adverse Events (CTCAE). The secondary outcomes included the proportion of heart failure, ischemic heart disease (IHD), atrial fibrillation (AF), and hypertension. Observational or pooled studies, reviews, letters, editorials, animal studies, study protocols, case reports, meeting abstracts, replies from authors, and articles not published in English were excluded. Furthermore, studies that did not provide clear data regarding the frequency of AEs were also excluded. References from all included papers were thoroughly examined to identify further pertinent studies.

### Data extraction

Two authors extracted data independently, including the first author’s name, publication year, study design, and demographic characteristics (such as age range and sample size), studied medications, treatment dosage, type of AEs, and their frequency. When the final report for AEs was not available, initial results were utilized. Regarding some data that has not been published, we received information directly from pharmaceutical companies [20, 21]. All discrepancies were resolved by consensus with co-authors.

### Risk of bias assessment

Study quality and risk of bias were evaluated using the Risk-of-Bias tool version 2 (RoB2) as outlined in the Cochrane Handbook for Systematic Reviews of Interventions (Supplementary Fig. 1) [22]. The Risk-of-Bias assessments of each study were conducted independently by two authors.

### Statistical analyses

#### Meta-analysis

Forest plots with risk ratios (RRs) were utilized to assess the association between ARPI plus ADT and various cardiovascular events including cardiac disorder, heart failure, IHD, AF, and hypertension, in comparison to SOC. The presence of heterogeneity among the outcomes of included studies in this meta-analysis was evaluated using Cochran’s Q test. In instances of significant heterogeneity (*p*-value of　<0.05 in Cochran’s Q test), we tried to investigate and explain the heterogeneity. Due to the likely heterogeneity arising from different disease states, we used a random-effects model to estimate RRs. To evaluate the presence of publication bias, funnel plots were used (Supplementary Fig. 2). In case more than ten studies were included, Egger’s test was also performed (Supplementary Fig. 3). All analyses were carried out with R version 4.3.0 (R Foundation for Statistical Computing, Vienna, Austria), and the statistical significance level was set at *p* < 0.05.

#### Network meta-analysis (NMA)

Network meta-analysis (NMA) was used for the simultaneous comparison of AEs in multiple treatment strategies and pooling of direct and indirect evidence. For each endpoint, network forest plots were generated [23, 24]. For the assessment of AEs, arm-based analyses were performed to estimate the RR of the AEs and 95% credible interval (CI) from the available raw data presented in the included articles. The relative effects were presented as RRs and 95% CIs. In addition, we estimated the relative ranking of the different treatments for each outcome using the surface under the cumulative ranking (SUCRA) [23]. All statistical analyses were performed using R version 4.3.0 (R Foundation for Statistical Computing, Vienna, Austria).

## Results

### Study selection and characteristics

**Fig. 1:**
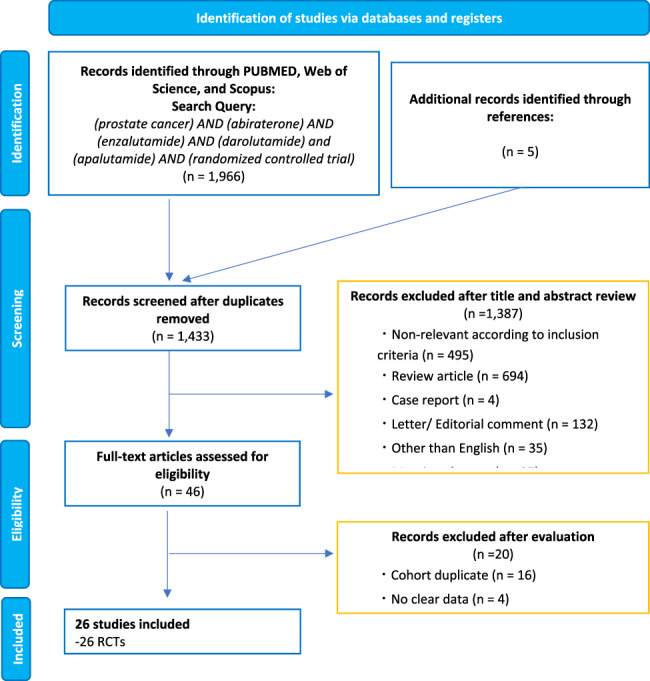
The preferred reporting items for systematic reviews and meta-analyses (PRISMA) flow chart, detailing the article selection process. PRISMA preferred reporting items for systematic reviews and meta-analyses.

Following our selection criteria, we identified 26 RCTs (Fig. 1) comprising 20,482 patients for meta-analyses and NMAs: three in the nmCRPC state [10, 14, 15], ten in the mCRPC state [9, 11–13, 25–30], two in the CRPC state [31, 32], eight in the metastatic/advanced HSPC state [3–7, 33–35], and three in the BCR state [36–38]. The median age of the patients ranged from 64 to 77 years, the median follow-up period ranged from 3.9 to 96 months, while the median duration of ARPI exposure was between 3.8 and 58 months. Some studies also included the concurrent use of prednisone or nonsteroidal antiandrogens in addition to ADT as shown in Table 1. Most studies except for NCT02294461 [26], ENABLE [31], and NCT02125357 [25] excluded patients who had suffered clinically significant heart disease and uncontrolled hypertension. The baseline characteristics of the studies can be found in Table 1.

**Table 1:** Study demographics of 26 RCTs included in the analyses.

Study/Author	Year	Treatment 1	Treatment 2	Disease status	Median follow-up	Median duration of exposure	Median age	ECOG PS (%)	No. of patients	No. of patients (T1)	No. of patients (T2)
ARAMIS Fizazi et al.	2019/2020	Darolutamide 600 mg + ADT	Placebo + ADT	nmCRPC	29	25.8	T1: 74(48–95) T2: 74(50–92)	T1 0: 68 1: 32 T2 0: 71 1: 29	1508	954	554
SPARTAN Smitih et al.	2018/2021	Apalutamide 240 mg+ ADT	Placebo + ADT	nmCRPC	52	32.9	T1: 74(48–94) T2: 74(52–97)	T1 0: 77 1: 23 T2 0: 78 1: 22	1201	803	398
PROSPER Hussain et al. Sternberg et al.	2018/2020	Enzalutamide 160 mg + ADT	Placebo + ADT	nmCRPC	48	33.9	T1: 74(50–95) T2: 73(53–92)	T1 0: 80 1: 20 Missing: <1 T2 0: 82 1: 18 Missing: <1	1395	930	465
PREVAIL Beer et al. Armstrong et al.	2014/2020	Enzalutamide 160 mg + ADT	Placebo + ADT	mCRPC	69	17.7	T1: 72(43–93) T2: 71(42–93)	T1 0: 67 1: 33 T2 0: 69 1: 31	1715	871	844
COU-AA-301 de Bono et al. Fizazi et al.	2011/2012	Abiraterone 1000 mg + predonisone10mg + ADT	Placebo + Predonisone10mg + ADT	mCRPC	20.2	7.4	T1: 69(42–95) T2: 69(39–90)	T1 0 or 1: 90 2: 10 T2 0 or 1: 89 2: 11	1185	791	394
COU-AA-302 Ryan et al.	2013/2015	Abiraterone 1000 mg + predonisone10mg + ADT	Placebo + Predonisone10mg + ADT	mCRPC	49.2	13.8	T1: 71(44–95) T2: 70(44–90)	T1 0: 76 1: 24 T2 0: 76 1: 24	1082	542	540
AFFIRM Scher et al.	2012	Enzalutamide 160 mg + ADT	Placebo + ADT	mCRPC	14.4	8.3	T1: 69(41–92) T2: 69(49–89)	T1 0 or 1: 91 2: 9 T2 0 or 1: 92 2: 8	1199	800	399
ARCHES Armstrong et al.	2019/2022	Enzalutamide 160 mg + ADT	Placebo + ADT	mHSPC	44.6	40.2	T1: 70(46–92) T2: 70(42–92)	T1 0: 78 1: 22 T2 0: 77 1: 23	1146	572	574
TITAN Chi et al.	2019/2021	Apalutamide 240 mg + ADT	Placebo + ADT	mHSPC	44	32.9	T1: 69(45–94) T2: 68(43–90)	T1 0: 62 1: 38 2: 0 T2 0: 66 1: 34 2: <1	1051	524	527
LATITUDE Fizazi et al.	2017/2019	Abiraterone 1000 mg + predonisone5mg + ADT	Placebo + Predonisone5mg + ADT	mHSPC	51.8	25.8	T1: 68(38–89) T2: 67(33–92)	T1 0-1: 96 2: 4 T2 0-1: 97 2: 3	1199	597	602
STAMPEDE (Arm G) James et al.	2017/2022	Abiraterone 100 mg + predonisone5mg + ADT	ADT	Advanced HSPC	73	23.7	T1: 67(39–84) T2: 67(42–85)	T1^a^ 0: 78 1-2: 22 T2 0: 78 1-2: 22	1908	948	960
ENZAMET Davis et al. Sweeney et al.	2019/2023	Enzalutamide 160 mg + ADT	Standard nonsteroidal antiandrogen + ADT	mHSPC	68	58	T1: 69.2(IQR: 63.2–74.5) T2: 69.0(IQR: 63.6–74.5)	T1 0: 72 1: 27 2: 1 T2 0: 72 1: 27 2: 1	1121	563	558
PEACE-1 Fizazi et al.	2022	Abiraterone 1000 mg + predonisone5mg + ADT	ADT	mHSPC	52.8	33.2	T1: 67(37–94) T2: 66(43–87)	NA	463	226	237
NCT01695135 Sun et al.	2016	Abiraterone 1000 mg + predonisone10mg + ADT	Placebo + Predonisone10mg + ADT	mCRPC	12.9	7.5	T1: 68.2 ± 8.30 T2: 67.7 ± 7.75	T1 0-1: 92 2: 8 T2 0-1: 93 2: 7	214	143	71
TERRAIN Shore et al.	2016	Enzalutamide 160 mg + ADT	Bicalutamide 50 mg + ADT	mCRPC	Enzalutamide: 20.0 Bicalutamide: 16.7	11.7	T1: 71(50–96) T2: 71(48–91)	T1 0: 71 1: 29 T2 0: 76 1: 24	372	183	189
NCT02294461 Pu et al.	2022	Enzalutamide 160 mg + ADT	Placebo + ADT	mCRPC	Enzalutamide: 34 Placebo: 80	6.6	T1: 71(51–89) T2: 71(50–88)	T1 0: 57 1: 43 T2 0: 65 1: 35	388	198	190
ENABLE Izumi et al.	2022	Enzalutamide 160 mg + ADT	Abiraterone 1000 mg + Prednizone5mg + ADT	CRPC	21.5	N.A.	T1: 75.7(70.2) T2: 77.4(71.8–81.5)	T1 0: 77 1: 21 Missing: 2 T2 0: 72 1: 25 Missing: 3	184	92	92
NCT02125357 Khalaf et al.	2019	Abiraterone 1000 mg + predonisone10mg + ADT	Enzalutamide 160 mg + ADT	mCRPC	22.8	N.A.	T1: 72.9(51.3–93.3) T2: 77.6(49.3–94.1)	T1 0-1: 88 T2 0-1: 78	202	101	101
LACOG0415 Maluf et al.	2021	Abiraterone 1000 mg + predonisone10mg + ADT	Apalutamide 240 mg	Advanced HSPC	14	N.A.	T1: 69(51–85) T2: 69.5(53–88)	T1 0-1: 98 T2 0-1: 100	84	42	42
NCT01591122 Ye et al.	2017	Abiraterone 1000 mg + predonisone5mg + ADT	Placebo + prednisone5mg + ADT	mCRPC	3.9	3.8	T1: 69.7 ± 8.72 T2: 70.8 ± 8.64	T1 0: 51 1: 49 T2 0: 52 1: 48	313	157	156
STAMPEDE (Arm J) Attard et al.	2023	Abiraterone 1000 mg + predonisone 5 mg + ADT	ADT	mHSPC	96	N.A.	T1: 67(42–85) T2: 67(39–84)	T1^a^ 0: 75 1: 24 2: 1 T2 0: 74 1: 25 2: 1	1000	498	502
STRIVE Penson et al.	2016	Enzalutamide 160 mg + ADT	Bicalutamide 50 mg + ADT	CRPC	T1: 16.7 months T2: 16.8 months	T1: 14.7 months T2: 8.4 months	T1: 72 (46–92) T2: 74 (50-91)	T1 0: 74.7 1: 25.3 T2 0: 73.2 1: 26.8	396	198	198
HEAT Ternov et al.	2022	Enzalutamide 160 mg + ADT	Abiraterone 1000 mg + Prednizone10mg + ADT	mCRPC	NA	NA	T1: 75 (51-88) T2: 77 (54–87)	T1 0: 69 1: 31 T2 0: 68 1: 32	169	84	85
EMBARK Freedland et al.	2023	Enzalutamide (160 mg) +ADT	Placebo + ADT	BCR	60.7	38.7 months (range: 0.1-88.9)	T1: 69 (51–87) T2: 70 (50–92)	T1 0: 92.4 1: 7.3 >1: 0.3 T2 0: 93.9 1: 5.9 Missing: 0.3	707	353	354
NCT01786265 Spetsieris et al.	2021	Abiraterone (1000 mg) + predonine (5 mg) + ADT	ADT	BCR	64.4 months	NA	T1: 65 (44–80) T2: 65 (42–85)	T1: median 0 (range 0-1) T2: median 0 (range 0-1)	197	99	98
NCT01751451 Autio et al.	2021	Abiraterone (1000 mg) + predonine (10 mg)	ADT	BCR	NA	NA	T1: 65 (53–74) T2: 64 (43–85)	T1 0: 95 1: 5 T2 0: 93 1: 7	83	41	42

*RCT* randomized controlled trial, *SD* standard deviation, *T* treatment arm, *C* control arm, *ADT* androgen deprivation therapy, *nmCRPC* non-metastatic castration-resistant prostate cancer, *mCRPC* metastatic castration-resistant prostate cancer, *mHSPC* metastatic hormone-sensitive prostate cancer, *CRPC* castration-resistant prostate cancer, *HSPC* castration sensitive prostate cancer, *N.A.* not applicable.

^a^WHO PS.

### Assessment of risk of bias and quality of study

The risk of bias judgments of each domain for each included study is summarized in Supplementary Fig. 1. All included studies had a low or moderate risk of bias owing to the nature of RCTs. The quality assessment of this meta-analysis was performed according to the AMSTAR2 checklist; overall confidence in the results of this review was “High” (Supplementary Appendix 1) [18]. Funnel plots of each analysis are depicted in Supplementary Fig. 2.

### Cardiac disorder

#### Meta-analysis of ARPI + ADT vs. SOC

These analyses were restricted to studies involving abiraterone and enzalutamide among ARPIs due to data availability. As shown in Table 2, ARPIs were associated with a statistically significant increase in both any grade (RR: 1.39, 95% CI: 1.10–2.22, *p* < 0.01) and grade ≥3 (RR: 1.74, 95% CI: 1.13–2.68, *p* = 0.01) cardiac disorder. Significant differences in disease state were observed for any grade cardiac disorder (*p* = 0.03) in mCRPC (RR: 1.19, 95% CI: 0.91–1.54) and mHSPC (RR: 1.75, 95% CI: 1.39–2.22). No significant difference was observed at grade ≥3 cardiac disorder (*p* = 0.6) (Supplementary Table 2). Cochran’s Q test revealed no significant heterogeneity in the analyses.

**Table 2:** Summary of meta-analysis on cardiovascular events with ARSIs.

		Data summary of included studies	Pooled RR (95% CI), *p*-value	Cochran’s Q test
Cardiac disorder	Any grade	5 studies 5786 patients	1.39 (1.10–2.22), *p* < 0.01	*p* = 0.06
	Grade ≥3	7 studies 6546 patients	1.74 (1.13–2.68), *p* = 0.01	*p* = 0.06
Heart failure	Any grade	9 studies 11,255 patients	1.60 (0.96–2.67), *p* = 0.07	*p* > 0.9
	Grade ≥3	7 studies 8988 patients	2.49 (1.05–5.91), *p* = 0.04	*p* > 0.9
IHD	Any grade	6 studies 7215 patients	2.36 (1.53–3.65), *p* < 0.001	*p* = 0.03
	Grade ≥3	5 studies 5820 patients	2.04 (0.91–4.62), *p* = 0.09	*p* < 0.01
AF	Any grade	9 studies 9412 patients	1.41 (1.02–1.94), *p* = 0.04	*p* = 0.5
	Grade ≥3	7 studies 7028 patients	2.15 (1.14–4.07), *p* = 0.02	*p* > 0.9
Hypertension	Any grade	19 studies 17,180 patients	1.68 (1.38–2.05), *p* < 0.001	*p* < 0.001
	Grade ≥3	16 studies 15,868 patients	2.06 (1.67–2.54), *p* < 0.01	*p* = 0.1

*ARSI* androgen receptor signaling inhibitor, *RR* risk ratio, *CI* Confidence Interval, *IHD* Ischemic heart disease, *AF* atrial fibrillation

#### Network meta-analysis

As shown in Table 3, abiraterone increased the risk of both any (RR: 1.48, 95% CI: 1.05–2.08) and ≥3 (RR: 2.40, 95% CI: 1.42–4.06) grade cardiac disorder compared to SOC. On the other hand, enzalutamide demonstrated no statistically significant difference in either case (any grade cardiac disorder: RR: 1.24, 95% CI: 0.80–1.92; grade ≥3 cardiac disorder: RR: 1.25, 95% CI: 0.73–2.13).　Based on the SUCRA analysis of treatment rankings, abiraterone was ranked highest for its association with the incidence of both any and grade ≥3 cardiac disorder. Cochran’s Q test revealed significant heterogeneity for any grade cardiac disorder (*p* = 0.03). Sensitivity analysis detected the AFFIRM study [9] as the cause of significant heterogeneity (Supplementary Fig. 6).

**Table 3:** Summary of NMA on cardiovascular events with ARSIs.

		Cardiac disorder		Heart failure		IHD		AF		Hypertension	
		Any grade	Grade ≥3	Any grade	Grade ≥3	Any grade	Grade ≥3	Any grade	Grade ≥3	Any grade	Grade ≥3
Data summary of included studies		5 studie 5 comparisons 5786 patients	7 studie 7 comparisons 6546 patients	10 studies 10 comparisons 11,424 patients	8 studies 8 comparisons 9157 patients	6 studies 6 comparisons 7215 patients	5 studies 5 comparisons 5820 patients	11 studies 11 comparisons 9765 patients	9 studies 9 comparisons 7,381 patients	23 studies 23 comparisons 17,819 patients	20 studies 20 comparisons 16,507 patients
SOC	RR (95% CI)	Ref.	Ref.	Ref.	Ref.	Ref.	Ref.	Ref.	Ref.	Ref.	Ref.
	SUCRA value	91%	90%	80%	85%	99%	86%	85%	88%	76%	75%
Abi	RR (95% CI)	1.48 (1.05–2.08)	2.40 (1.42–4.06)	1.77 (0.52–6.02)	2.93 (0.51–16.86)	NA	NA	1.51 (0.96–2.38)	2.07 (0.85–5.08)	1.70 (1.32–2.19)	2.19 (1.77–2.70)
	SUCRA value	13%	2%	37%	37%			27%	37%	26%	19%
Apa	RR (95% CI)	NA	NA	1.33 (0.42–4.17)	1.92 (0.26–14.46)	2.32 (0.97–5.54)	2.08 (0.46–9.41)	1.31 (0.63–2.71)	1.55 (0.45–5.26)	0.88 (0.53–1.46)	0.63 (0.31–1.29)
	SUCRA value			54%	53%	25%	32%	47%	59%	85%	96%
Dar	RR (95% CI)	NA	NA	2.09 (0.77–5.66)	4.65 (0.25–88.20)	NA	NA	NA	NA	1.19 (0.57–2.49)	1.47 (0.77–2.82)
	SUCRA value			27%	30%					57%	49%
Enz	RR (95% CI)	1.24 (0.80–1.92)	1.25 (0.73–2.13)	1.40 (0.62–3.16)	2.32 (0.74–7.28)	2.38 (1.32–4.30)	2.02 (0.62–6.63)	1.37 (0.74–2.53)	3.17 (1.05–9.58)	2.08 (1.61–2.70)	2.30 (1.82–2.92)
	SUCRA value	46%	58%	53%	44%	26%	31%	41%	15%	5%	12%
Ranking		SOC>Enz>Abi	SOC>Enz>Abi	SOC>Apa>Enz>Abi>Dar	SOC>Apa>Enz>Abi>Dar	SOC>Enz>Apa	SOC>Apa>Enz	SOC>Apa>Enz>Abi	SOC>Apa>Abi>Enz	Apa>SOC>Dar>Abi>Enz	Apa>SOC>Dar>Abi>Enz
Cochran’s Q test		*p* = 0.03	*p* = 0.2	*p* = 0.9	*p* = 0.9	*p* = 0.01	*p* < 0.01	*p* = 0.4	*p* > 0.9	*p* < 0.001	*p* = 0.5

*NMA* network meta-analysis, *RR* risk ratio, *CI* Confidence Interval, *IHD* ischemic heart disease, *AF* atrial fibrillation, *Ref* reference, *NA* not available, *SOC* standard of care, *Abi* abiraterone, *Enz* enzalutamide, *Apa* apalutamide, *Dar* darolutamide

### Heart failure

#### Meta-analysis of ARPI + ADT vs. SOC

ARPIs plus ADT were associated with an increased risk of grade ≥3 heart failure (RR: 2.49, 95% CI: 1.05–5.91, *p* = 0.04). For any grade heart failure, statistical significance was not reached (RR: 1.60, 95% CI: 0.96–2.67, *p* = 0.07) (Table 2). No significant differences in risk were noted between the three disease states for either any grade or grade ≥3 heart failure (both *p* = 0.6) (Supplementary Table 2). Cochran’s Q test revealed no significant heterogeneity in the analyses.

#### Network meta-analysis

None of the ARPIs demonstrated significant RR for both any grade and grade ≥3 heart failure (Table 3). Based on the SUCRA analysis of treatment rankings, darolutamide presented the highest risk of both any grade (27%) and grade ≥3 (30%) heart failure, followed by abiraterone. We did not find any significant heterogeneity for both results.

### Ischemic heart disease (IHD)

#### Meta-analysis of ARPI + ADT vs. SOC

These analyses were restricted to studies involving abiraterone and enzalutamide among ARPIs due to data availability. ARPIs plus ADT were associated with a statistically significant increase in the risk of any grade IHD (RR: 2.36, 95% CI: 1.53–3.65, *p* < 0.001). However, the increase in risk did not reach statistical significance for grade ≥3 IHD (RR: 2.04, 95% CI: 0.91–4.62, *p* = 0.09). Significant differences in disease state were observed at any grade IHD (*p* = 0.03), with BCR status showing a lower RR (RR: 0.95, 95% CI: 0.50–3.65) compared to others (Supplementary Table 2). Cochran’s Q test revealed significant heterogeneity for grade ≥3 IHD (*p* < 0.01). Sensitivity analysis revealed the PREVAIL study [13] as a source of this heterogeneity (Supplementary Fig. 5).

#### Network meta-analysis

As shown in Table 3, enzalutamide showed a significantly higher incidence of any grade IHD compared to SOC (RR 2.38, 95% CI: 1.32–4.30). In contrast, apalutamide and enzalutamide did not demonstrate significant RR for grade ≥3 IHD. According to SUCRA analysis for treatment rankings, there is minimal difference in both any grade (apalutamide: 25%, enzalutamide: 26%) and grade ≥3 (apalutamide: 32%, enzalutamide: 31%) IHD. Significant heterogeneity was found for both any (*p* = 0.01) and grade ≥3 (*p* < 0.01) IHD. Sensitivity analyses identified the EMBARK study [36] as the source of significant heterogeneity for any grade IHD and the PREVAIL study [13] for grade ≥3 IHD (Supplementary Fig. 6).

### Atrial fibrillation (AF)

#### Meta-analysis of ARPI + ADT vs. SOC

ARPIs plus ADT were associated with a statistically significant increase in the risk of both any grade (RR: 1.41, 95% CI: 1.02–1.94, *p* = 0.04) and grade ≥3 (RR: 2.15, 95% CI: 1.14–4.07, *p* = 0.02) AF (Table 3). No significant differences in risk were noted between the various disease states for either any grade (*p* = 0.9) or grade ≥3 AF (*p* = 0.8) (Supplementary Table 2). No heterogeneity was observed in any of the analyses.

#### Network meta-analysis

Enzalutamide demonstrated a significant increase in the risk of grade ≥3 AF (RR: 3.17, 95% CI: 1.05–9.58), but not any grade AF. No other type of ARPI showed a significantly higher incidence of both any grade and grade ≥3 AF (Table 3). According to the SUCRA analysis for treatment ranking, abiraterone had the highest risk for any grade AF (27%), and enzalutamide for grade ≥3 AF (15%). No heterogeneity was observed in any of the analyses.

### Hypertension

#### Meta-analysis of ARPI + ADT vs. SOC

ARPIs plus ADT were associated with a statistically significant increase in the risk of hypertension, both for all grades (RR: 1.68, 95% CI: 1.38–2.05, *p* < 0.001) and grade ≥3 (RR: 2.06, 95% CI: 1.67–2.54, *p* < 0.001) as shown in Table 2. No significant differences in disease states were observed at any grade (*p* = 0.09) and grade ≥3 hypertension (*p* = 0.8) (Supplementary Table 2). The Cochran’s Q test revealed significant heterogeneity in the analysis for any grade hypertension (*p* < 0.01). Subgroups based on disease state and sensitivity analyses were unable to identify the source of significant heterogeneity for hypertension of any grade (Supplementary Table 2 and Supplementary Fig. 5). However, the funnel plot exhibited symmetry, and Egger’s test did not indicate significant publication bias (F (1, 17) = 1.8, *p* = 0.2) (Supplementary Figs. 2, 3).

#### Network meta-analysis

Abiraterone and enzalutamide increased the risk significantly in both any grade (abiraterone: RR 1.70; 95% CI 1.32–2.19; enzalutamide: RR 2.08; 95% CI 1.61–2.70) and grade ≥3 hypertension (abiraterone: RR 2.19; 95% CI 1.77–2.70; enzalutamide: RR 2.30; 95% CI: 1.82–2.92) compared to SOC as shown in Table 3. Based on the SUCRA analysis of treatment rankings, enzalutamide had the highest risk of both any grade and grade ≥3 hypertension, followed by abiraterone. The Cochran’s Q test revealed significant heterogeneity for any grade hypertension (*p* < 0.001). The subgroup analyses based on disease state and sensitivity analyses were unable to identify the source of significant heterogeneity for any grade hypertension (Supplementary Table 3 and Supplementary Fig. 6).

## Discussion

This is the first meta-analysis and NMA to comprehensively synthesize and compare the incidence of cardiovascular events in advanced PCa patients treated with ARPIs. Our study presents several key findings. First, our meta-analyses indicate that adding ARPIs to ADT increases the risk of various cardiovascular events compared to SOC. Second, our NMAs reveal that abiraterone plus ADT increases the risk of cardiac disorder and hypertension compared to SOC. Third, enzalutamide plus ADT was found to increase the risk of IHD and hypertension compared to SOC based on our NMAs.

Our analyses revealed that adding ARPIs to ADT increases the risk of cardiac disorder by 39% compared to SOC, elevating the risk of high-grade toxicity by 74%. In addition to these overall trends, we observed notable increases in the risk of specific any grade cardiac disorders: IHD up to 136% (104% for grade ≥3), AF up to 41% (115% for grade ≥3), and hypertension up to 68% (106% for grade ≥3). Although the increase in the risk of any grade heart failure associated with ARPIs plus ADT was not statistically significant, reaching up to 60%, a significant increase was observed for grade ≥3 heart failure at 149%. It should be noted that the majority of the RCTs included in our analyses excluded patients with pre-existing significant heart disease and uncontrolled hypertension. Therefore, the actual incidence of cardiovascular events in a broader patient population may be even higher for both SOC and ARPI plus ADT.　This underscores the importance of real-world data, which often includes all patients to obtain a more comprehensive and realistic understanding of the cardiovascular safety profile of ARPIs. For example, in a real-world data study comprising 4962 mCRPC patients ARPIs were associated with a threefold increase in the risk of major adverse cardiovascular events (HR: 3.15, 95% CI: 2.03–4.89), an almost fivefold increase in risk of acute coronary syndrome (HR: 4.94, 95% CI: 2.36–10.33) and close to threefold increase in the risk of heart failure (HR: 2.83, 95% CI: 1.53–5.25) [39].

In our NMAs, abiraterone was found to significantly increase the risks of cardiac disorder (48%) and hypertension (70%). Interestingly, abiraterone was not associated with any changes in the risk of heart failure and AF. Abiraterone inhibits CYP17, reducing cortisol but stimulating adrenocorticotropic hormone (ACTH) levels, which in turn leads to hypertension [40]. To mitigate this and other AEs, corticosteroids are coadministered to control adrenocorticotropic hormone release. Nevertheless, despite corticosteroid co-administration, hypertension was observed in 3–70% of cases across RCTs [5, 6, 11, 12, 25, 28–31, 36–38]. Hypertension is a risk factor for several types of cardiac maladies such as heart failure, IHD, AF, and valvular disease [41]. Real-world data represented by Bretagne et al. [42], demonstrated that ADT plus abiraterone increases the risk of hypertension (Odds ratio [OR]: 1.8, 95% CI: 1.5–2.0) and heart failure (OR: 1.5, 95% CI: 1.3–1.7) compared to ADT alone. Although our analyses did not indicate an elevated risk for heart failure and AF with abiraterone plus ADT, it should be noted that long-term uncontrolled hypertension can potentially lead to these consequences.

We found that adding enzalutamide to ADT increases the risk of IHD by 138% and hypertension by 108% compared to SOC. Due to data availability limitations, our analysis of IHD was limited to only apalutamide and enzalutamide. Within these limitations, our findings indicated that enzalutamide was almost equivalent to apalutamide in terms of IHD risk. Regarding myocardial infarction (MI), a condition often considered a severe manifestation of IHD, enzalutamide did not show a significant increase in MI (RR: 1.53, 95% CI: 0.64–3.62), but had a higher likelihood at SUCRA ranking for MI (44%) compared to abiraterone (76%) (Supplementary Table 5). The impact of enzalutamide on the cardiovascular system may be attributed to its role in blocking androgen activity, which could explain its cardiovascular effects. Furthermore, enzalutamide has the potential to trigger apoptosis in cardiovascular cells and provoke oxidative stress, contributing to the onset of cardiovascular diseases [43]. Real-world data, by Liu et al. [43] revealed that abiraterone carried a higher risk of MI than enzalutamide (HR: 2.43, 95% CI: 2.03–2.91, *p* < 0.001). Similarly, Conver et al. [44] also found a higher risk associated with abiraterone compared to enzalutamide (HR: 2.04, 95% CI: 1.16–3.69). The discrepancy in the MI results of this study is due to the real-world nature which included all patients and did not use the selective criteria of RCTs. Moreover, the statistical power of these RCTs may have been limited because of the low incidence of IHD and MI.

Adding apalutamide to ADT was not found to increase the risk of heart failure, IHD, AF, and hypertension. However, it had the highest risk of IHD, followed by enzalutamide. Additionally, the RR for MI was significantly higher than that of SOC (RR: 7.74, 95% CI: 1.00–60.06), with apalutamide ranking highest for MI risk in the SUCRA ranking (Supplementary Table 5). Liu et al. [43] analyzed real-world data to assess non-fatal MI and found apalutamide had a significantly higher risk compared to enzalutamide (OR: 2.26, 95% CI: 1.53–3.32). The constrained quantity of studies conducted on apalutamide could account for the observation that apalutamide did not exhibit a statistically significant increase in the risk of cardiovascular events except MI. Due to the similarity of apalutamide and enzalutamide, it is crucial to pay adequate caution regarding cardiovascular events.

For darolutamide, our analyses were limited to heart failure and hypertension due to the lack of data arising only from the ARAMIS trial [10]. Within this limited data, there was no significant increase in heart failure and hypertension with the addition of darolutamide to ADT compared to SOC. Due to the limitations of data availability, the safety profile of darolutamide remains unclear. Although the ARASENS trial [45] demonstrated a higher incidence of hypertension with darolutamide added to docetaxel + ADT compared to docetaxel + ADT (RR: 1.48, 95% CI: 1.09–2.01), no significant increase was observed for cardiac disorder, coronary artery disorder, and heart failure. Considering the outcomes of our analyses and the ARASENS trial [45], darolutamide seems to be one of the safer options among the ARPIs when it comes to cardiac AEs. However, there is uncertainty around the safety profile of darolutamide, and more data are needed to draw definitive conclusions. The data from the ARANOTE trial [46], which assesses darolutamide + ADT vs. ADT alone in mHSPC patients, are eagerly awaited.

The present study has several limitations that need to be considered. First, this meta-analysis and NMA included RCTs that varied significantly in terms of patient populations, disease states, inclusion and exclusion criteria, and methods of reporting. Therefore, we conducted subgroup analyses across the different disease states. Despite this, our results need to be interpreted with much caution due to the limited number of events and potential sources of bias as outlined above. In addition, NMAs have a limited value in comparing heterogeneous data and can only be considered as an information source for proper patient selection. No statistical adjustment can substitute a direct comparison of each treatment in an RCT and is, therefore, only to be considered as hypothesis-generating. Second, the follow-up duration and exposure duration to the drugs varied across the included studies, potentially leading to inconsistencies in the reporting of AEs. It should be noted that extended periods of treatment and observation may intrinsically elevate the likelihood of AE occurrence, therefore impacting the inter-study comparability. Third, the unexplained heterogeneity for any grade of hypertension serves as a limitation and suggests caution in the interpretation of our meta-analysis results. Fourth, due to inconsistencies in how AEs were reported across studies, we attempted to standardize the criteria for comparison. However, this led to data limitations, reducing the number of studies that could be included in the analysis for specific AEs except for hypertension. Finally, it should be noted that ADT in itself is known to pose a risk for cardiovascular events [47]. Additionally, in some studies, the use of other agents, such as first-generation antiandrogens, was permitted within the SOC group. It should also be noted that the ARCHES [3], ENZAMET [4], and TITAN [7] trials allowed the use of docetaxel as well after randomization, which could further increase the risk of cardiovascular events [48–50], potentially affecting the study outcomes and limiting the generalizability of our findings.

## Conclusion

In our investigation, we observed that adding ARPIs to ADT elevates the likelihood of cardiovascular events in PCa patients compared to SOC. NMAs highlighted distinct cardiovascular risk profiles for various ARPIs. Abiraterone correlated with increased risks of cardiac disorders and hypertension, while enzalutamide showed elevated risks of IHD and hypertension. These findings emphasize the imperative for meticulous patient selection, counseling, optimization, and monitoring during the administration of these therapies. Additionally, it is important to note that cardiac AEs may be even higher in real world.

## Supplementary information

Supplementary Information
