# Publisher Correction: Cardiovascular events among men with prostate cancer treated with androgen receptor signaling inhibitors: a systematic review, meta-analysis, and network meta-analysis

**DOI:** 10.1038/s41391-024-00902-3

**Published:** 2024-10-07

**Authors:** Akihiro Matsukawa, Takafumi Yanagisawa, Mehdi Kardoust Parizi, Ekaterina Laukhtina, Jakob Klemm, Tamás Fazekas, Keiichiro Mori, Shoji Kimura, Alberto Briganti, Guillaume Ploussard, Pierre I. Karakiewicz, Jun Miki, Takahiro Kimura, Pawel Rajwa, Shahrokh F. Shariat

**Affiliations:** 1 Department of Urology, Comprehensive Cancer Center, Medical University of Vienna, Vienna, Austria; 2 Department of Urology, The Jikei University School of Medicine, Tokyo, Japan; 3 Department of Urology, Shariati Hospital, Tehran University of Medical Science, Tehran, Iran; 4 Department of Urology, University Medical Center Hamburg-Eppendorf, Hamburg, Germany; 5 Department of Urology, Semmelweis University, Budapest, Hungary; 6 Unit of Urology, Urological Research Institute, IRCCS San Raffaele Scientific Institute, Milan, Italy; 7 Vita-Salute San Raffaele University, Milan, Italy; 8 Department of Urology, La Croix du Sud Hospital, Quint Fonsegrives, France; 9 Cancer Prognostics and Health Outcomes Unit, Division of Urology, University of Montréal Health Center, Montréal, QC Canada; 10 Department of Urology, Medical University of Silesia, Zabrze, Poland; 11 Department of Urology, University of Texas Southwestern Medical Center, Dallas, TX USA; 12 Department of Urology, Weill Cornell Medical College, New York, NY USA; 13 Department of Urology, Second Faculty of Medicine, Charles University, Prague, Czechia; 14 Division of Urology, Department of Special Surgery, The University of Jordan, Amman, Jordan; 15 Karl Landsteiner Institute of Urology and Andrology, Vienna, Austria; 16 Research Center for Evidence Medicine, Urology Department Tabriz University of Medical Sciences, Tabriz, Iran

**Keywords:** Cancer therapy, Outcomes research

Correction to: *Prostate Cancer and Prostatic Diseases* 10.1038/s41391-024-00886-0, published online 5 September 2024

In Table 3 of this article, the data in column 1–7 were mistakenly listed. Please see the corrected table below.

Table 3 Summary of NMA on cardiovascular events with ARSIs.

**Table:** 

		Cardiac disorder		Heart failure		IHD		AF		Hypertension	
		Any grade	Grade ≥3	Any grade	Grade ≥3	Any grade	Grade ≥3	Any grade	Grade ≥3	Any grade	Grade ≥3
Data summary of included studies		5 studie 5 comparisons 5786 patients	7 studie 7 comparisons 6546 patients	10 studies 10 comparisons 11,424 patients	8 studies 8 comparisons 9157 patients	6 studies 6 comparisons 7215 patients	5 studies 5 comparisons 5820 patients	11 studies 11 comparisons 9765 patients	9 studies 9 comparisons 7,381 patients	23 studies 23 comparisons 17,819 patients	20 studies 20 comparisons 16,507 patients
SOC	RR (95% CI)	Ref.	Ref.	Ref.	Ref.	Ref.	Ref.	Ref.	Ref.	Ref.	Ref.
	SUCRA value	91%	90%	80%	85%	99%	86%	85%	88%	76%	75%
Abi	RR (95% CI)	1.48 (1.05–2.08)	2.40 (1.42–4.06)	1.77 (0.52–6.02)	2.93 (0.51–16.86)	NA	NA	1.51 (0.96–2.38)	2.07 (0.85–5.08)	1.70 (1.32–2.19)	2.19 (1.77–2.70)
	SUCRA value	13%	2%	37%	37%			27%	37%	26%	19%
Apa	RR (95% CI)	NA	NA	1.33 (0.42–4.17)	1.92 (0.26–14.46)	2.32 (0.97–5.54)	2.08 (0.46–9.41)	1.31 (0.63–2.71)	1.55 (0.45–5.26)	0.88 (0.53–1.46)	0.63 (0.31–1.29)
	SUCRA value			54%	53%	25%	32%	47%	59%	85%	96%
Dar	RR (95% CI)	NA	NA	2.09 (0.77–5.66)	4.65 (0.25–88.20)	NA	NA	NA	NA	1.19 (0.57–2.49)	1.47 (0.77–2.82)
	SUCRA value			27%	30%					57%	49%
Enz	RR (95% CI)	1.24 (0.80–1.92)	1.25 (0.73–2.13)	1.40 (0.62–3.16)	2.32 (0.74–7.28)	2.38 (1.32–4.30)	2.02 (0.62–6.63)	1.37 (0.74–2.53)	3.17 (1.05–9.58)	2.08 (1.61–2.70)	2.30 (1.82–2.92)
	SUCRA value	46%	58%	53%	44%	26%	31%	41%	15%	5%	12%
Ranking		SOC>Enz>Abi	SOC>Enz>Abi	SOC>Apa>Enz>Abi>Dar	SOC>Apa>Enz>Abi>Dar	SOC>Enz>Apa	SOC>Apa>Enz	SOC>Apa>Enz>Abi	SOC>Apa>Abi>Enz	Apa>SOC>Dar>Abi>Enz	Apa>SOC>Dar>Abi>Enz
Cochran’s Q test		*p* = 0.03	*p* = 0.2	*p* = 0.9	*p* = 0.9	*p* = 0.01	*p* < 0.01	*p* = 0.4	*p* > 0.9	*p* < 0.001	*p* = 0.5

*NMA* network meta-analysis, *RR* risk ratio, *CI* Confidence Interval, *IHD* ischemic heart disease, *AF* atrial fibrillation, *Ref* reference, *NA* not available, *SOC* standard of care, *Abi* abiraterone, *Enz* enzalutamide, *Apa* apalutamide, *Dar* darolutamide.

